# Association of skin autofluorescence with low bone density/osteoporosis and osteoporotic fractures in type 2 diabetes mellitus

**DOI:** 10.1111/1753-0407.13309

**Published:** 2022-09-04

**Authors:** Hongyan Liu, Guoqi Wang, Ting Wu, Jia Hu, Yiming Mu, Weijun Gu

**Affiliations:** ^1^ Department of Endocrinology The First Medical Center of Chinese PLA General Hospital Beijing China; ^2^ Department of Pediatrics The First Medical Center of Chinese PLA General Hospital Beijing China

**Keywords:** advanced glycation end products (AGEs), bone turnover, osteoporosis, osteoporotic fractures, type 2 diabetes mellitus (T2DM), 糖基化终产物(AGEs), 骨质疏松骨折, 骨质疏松, 2型糖尿病(T2DM), 骨转换

## Abstract

**Background:**

Advanced glycation end products (AGEs) that abnormally accumulate in diabetic patients have been reported to damage bone health. We aimed to investigate the association between skin autofluorescence (SAF)‐AGE_age_ (SAF − AGEs × age/100) and low bone density (LBD)/osteoporosis or major osteoporotic fractures (MOFs) in patients with type 2 diabetes mellitus (T2DM).

**Methods:**

This study was nested in the prospective REACTION (Risk Evaluation of Cancers in Chinese Diabetic Individuals) study and included 1214 eligible participants. SAF was used to measure skin AGEs (SAF‐AGEs). Fracture events were determined by an in‐person clinical follow‐up. Binary logistic regression analysis, linear regression analysis, and a restricted cubic spline nested in logistic models were used to test outcomes.

**Results:**

The overall prevalence of LBD/osteoporosis in middle‐aged or elderly T2DM patients was 35.7% (n = 434), and the overall incidence of MOFs was 10.5% (n = 116). Logistic analysis showed a significantly positive relationship between quartiles of SAF‐AGE_age_ and the risk of LBD/osteoporosis (odds ratio [OR] 2.02, 95% CI 1.34–3.03; OR 3.63, CI 2.44–5.39; and OR 6.51, CI 4.34–9.78) for the multivariate‐adjusted models, respectively. SAF‐AGE_age_ was associated with MOFs with a multivariate‐adjusted OR of 1.02 (CI 0.52–2.02), 2.42 (CI 1.32–4.46), and 2.70 (CI 1.48–4.91), respectively. Stratified analyses showed that SAF‐AGE_age_ was significantly associated with MOFs only in females, nonsmokers, nondrinkers, individuals with lower body mass index, and those without LBD/osteoporosis. Linear regression analyses showed that higher SAF‐AGEs were associated with a higher level of serum N‐terminal propeptide of type I procollagen (s‐PINP) and serum carboxy‐terminal cross‐linking peptide of type I collagen (s‐CTX), with a multivariate‐adjusted OR of 1.02 (CI 0.24–1.80) and 6.30 (CI 1.77–10.83), respectively.

**Conclusions:**

In conclusion, SAF‐AGE_age_ was positively associated with the prevalence of LBD/osteoporosis or MOFs in patients with T2DM. A positive association between SAF‐AGEs and the level of s‐PINP and s‐CTX was found.

## Graphical abstract

**Highlights**

- The association between skin autofluorescence advanced glycation end products (SAF‐AGE_age_) and low bone density/osteoporosis or major osteoporotic fractures was noteworthy in patients with type 2 diabetes mellitus (T2DM).
- SAF‐AGEs were positively associated with the level of serum N‐terminal propeptide of type I procollagen (s‐PINP) and serum carboxy‐terminal cross‐linking peptide of type I collagen (s‐CTX).
- To our knowledge, this is the first article that reports the association between SAF‐AGEs and bone turnover markers in patients with T2DM.

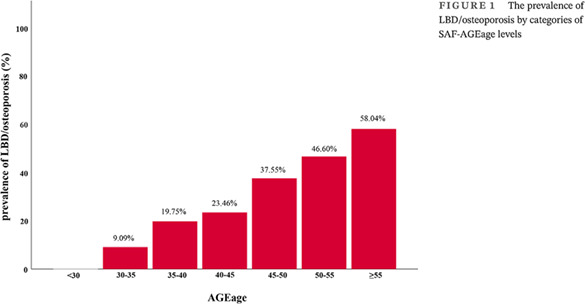

## 摘要

**背景:**

糖基化终末产物(AGEs)在糖尿病患者体内异常积聚，已有报道会损害骨健康。本研究旨在探讨皮肤自发荧光(SAF)‐AGE_age_(SAF‐AGEs×age/100)与2型糖尿病(T2DM)患者低骨密度(LBD)/骨质疏松或主要骨质疏松性骨折(MOF)的关系。

**方法:**

本研究整合在前瞻性 (中国糖尿病患者癌症风险评估) REACTION研究中，包括1214名符合条件的参与者。用SAF测量皮肤年龄(SAF‐AGE)。骨折事件通过临床随访来确定。使用二元Logistic回归分析, 线性回归分析和在Logistic模型中的限制立方样条法来检验结果。

**结果:**

中老年T2DM患者LBD/骨质疏松总患病率为35.7%(n=434)，MOFs总患病率为10.5%(n=116)。Logistic回归分析显示，SAF‐AGE_age_的四分位数与LBD/骨质疏松的风险呈显著正相关[OR 2.02，95%置信区间(CI)1.34~3.03；OR 3.63，CI 2.44~5.39；OR 6.51；CI 4.34~9.78]。SAF‐AGE_age_与MOF相关，多因素校正OR值分别为1.02；CI 0.52~2.02，OR 2.42；CI 1.32~4.46，OR 2.70；CI 1.48~4.91。分层分析显示，SAF‐AGE_age_仅在女性, 非吸烟者, 不饮酒者, 体重指数较低的个人以及没有LBD/骨质疏松的人中与MOF显著相关。线性回归分析显示，SAF‐AGEs越高，血清s‐PINP和s‐CTX水平越高，多因素校正后的OR值分别为1.02, CI 0.24~1.80和6.30, CI 1.77~10.83。

**结论:**

在T2DM患者中，SAF‐AGE_age_与LBD/骨质疏松或MOFs的患病率呈正相关。SAF‐AGEs与血清s‐PINP, s‐CTX水平呈正相关。

## 1 INTRODUCTION

Osteoporosis is an important complication of type 2 diabetes mellitus (T2DM) and the most common systemic bone disease. It is characterized by low bone mass and microstructural damage to bone tissue^1^ that manifests with increased bone fragility and an increased risk of fracture.^2^ Prior work has shown osteoporotic fractures are particularly common in the vertebral bodies.^3^ Osteoporosis‐related bone fractures pose one of the important risks for disability and death in the elderly and have thus received growing attention. It has been reported that around 40% of mainland Chinese T2DM patients have osteoporosis^4^ and are at a significantly higher risk of bone fracture than healthy individuals.^5^

Advanced glycation end products (AGEs) are stable and irreversible heterogeneous compounds that are formed nonenzymatically as a result of Maillard or browning reactions. Identified structures include pentosidine, carboxymethyllysine (CML), pyrraline, and crossline.^6^ AGEs, as the physiological products of the life cycle, accumulate abnormally in diabetic patients due to persistent hyperglycemia and other pathological changes. AGEs can affect the structures and function of proteins and cause tissue damage by trapping and cross‐linking structural and blood proteins directly or indirectly, especially collagens.^7^, ^8^ Type I collagen is a key component of the bone matrix. It has been demonstrated that in vitro, the synthesis and secretion of osteocalcin and type I collagen of osteoblasts decreased significantly when AGE‐bovine serum albumin (BSA) was added to the culture medium.^9^ Hein et al. reported that in osteoporotic patients compared with healthy subjects, the serum concentrations of pentosidine and CML are increased and that pentosidine may increase bone resorption of osteoclasts.^10^ The study by Choi D. H. et al found an association between increased serum pentosidine levels and reduced bone mineral density (BMD) or osteoporotic fractures in patients without DM.^11^

Skin autofluorescence (SAF) is a noninvasive method to measure skin AGEs (SAF‐AGEs). A close relationship has been demonstrated between the SAF‐AGEs value and that obtained via a skin biopsy.^12^, ^13^ Tabara et al found that SAF‐AGEs are associated with reduced BMD, low skeletal muscle mass, and weak muscle strength in the general population (with or without DM).^14^ The Rotterdam Study demonstrated that subjects with osteoporotic fractures had higher SAF‐AGE values.^15^

There are few studies investigating the relationship between BMD or osteoporotic fractures and AGEs in patients with T2DM. Given the importance of age on AGEs and osteoporosis or osteoporotic fractures, we used the age‐combined SAF‐AGE (SAF‐AGE_age_) index (SAF − AGEs × age/100) in the related investigation of AGEs and osteoporosis or osteoporotic fractures in our study. Our main aim was to investigate the association between SAF‐AGE_age_ and low bone density (LBD)/osteoporosis or osteoporotic fractures in patients with T2DM and to test how AGEs affect bone turnover. The influence of age on bone turnover markers is not known, so the SAF‐AGEs index instead of the SAF‐AGE_age_ index was used in the related study of AGEs and bone turnover.

## 2 METHODS

### 2.1 Study population and design

The present study was nested in the REACTION (Risk Evaluation of Cancers in Chinese Diabetic Individuals) study, an ongoing multicenter prospective cohort study designed to research the relationship between T2DM and prediabetes with the risk of cancer in the People's Republic of China; details have been described previously.^16^ The study protocol was approved by the Committee on Human Research at the Chinese People's Liberation Army (PLA) General Hospital, and written informed consent was obtained from all participants prior to participation. For our study, 6854 participants aged 40 years or older were recruited in central Beijing between September and December 2018. T2DM was diagnosed according to the 1999 World Health Organization (WHO) criteria. The exclusion criteria of our study were (1) a diagnosis of hyperparathyroidism, hyperthyroidism, end‐stage kidney disease, rheumatic disease (such as rheumatoid arthritis, systemic lupus erythematosus, etc.), psychiatric disorder, severe liver dysfunction, or cancers that might affect bone metabolism; (2) no diagnosis of T2DM; (3) current use of glucocorticoids, steroid hormones, antiepileptic drugs, chemotherapy drugs, antiviral drugs, or proton pump inhibitor use within 3 months; and (4) individuals without complete requisite data. Based on these criteria, the final cohort included 1214 eligible participants.

### 2.2 Measurement of SAF

A spectroscopy device (AGE Reader, Hefei Institutes of Physical Science, Chinese Academy of Sciences) was used to measure skin AGEs. The AGE Reader is a noninvasive instrument that quantifies AGEs based on their fluorescent properties (excitation is at 300–420 nm and emission at 420–600 nm). An excitation light source with a peak wavelength of 370 nm was used to illuminate a 1–4‐cm section of the forearm. Local creams or lotions were withheld for 12 h before the examination, and the participant was instructed to stay immobile during the acquisition time (around 30 s). The emitted and reflected light was measured by the AGE Reader. The AGE Reader software automatically calculated SAF based on the ratio between the emitted and reflected light. Three examinations were conducted by trained operators, and an average score was obtained for statistical analysis.

### 2.3 Measurement of BMD

BMD was measured via dual‐energy X‐ray absorptiometry (DXA) using a Lunar Prodigy DXA bone densitometer (GE Lunar Corp, Madison, Wisconsin) and expressed in g/cm^2^. The main measurement sites were the lumbar spine (L1–L4), total hip, and femoral neck. The distal radius was utilized if the three main sites proved difficult to measure. The scans were conducted by a single trained technician and assessed by a third specialist if necessary. T‐scores were calculated based on the measured BMD value and peak BMD value from normal young people of the same race and sex. According to the diagnostic criteria recommended by the WHO, osteoporosis was defined as a T‐score below −2.5, and LBD was defined as a T‐score between −1.0 and − 2.5.

### 2.4 Confirmation of major osteoporotic fractures

Follow‐up for fracture events was continued until 31 March 2022. Locations of osteoporotic fractures were classified as home or workplace/shopping mall. Reasons for osteoporotic fractures were classified as a “minor external force collision” or a “fall.” Sites of major osteoporotic fractures (MOFs) included the vertebra, hip, wrist, or humerus. All fracture events were diagnosed by clinicians and confirmed by radiographs in a grade A tertiary hospital.

### 2.5 Clinical examinations

Each individual was comprehensively examined including a detailed questionnaire, routine physical examinations, a standard 75‐g oral glucose tolerance test (OGTT)/100‐g steamed‐bread meal test (SBMT), and laboratory measurements. The content of the questionnaire included age, sex, smoking status, drinking status, eating habits, physical exercise, sleep, medical history, familial medical history, medication history, surgical history, etc. Current smoking was defined as smoking more than once daily within the last 3 months. Current drinking was defined as recent drinking of nearly/more than once a week. Eating habits mainly referred to daily salt intake and were classified as greater or less than 6 g. Familial medical history included that of first‐degree relatives. Height (cm), weight (kg), waist circumference (cm), and hip circumference (cm) were measured in a standard standing position wearing light clothing without shoes and recorded to one decimal point. Body mass index (BMI) was calculated as the weight (kg) divided by the height (m) squared (kg/m^2^). Waist to hip ratio (WHR) was calculated as waist circumference (cm) divided by hip circumference (cm). Blood pressure was measured in a standard sitting position, three times every 5 min, and an average value was used for statistical analysis.

### 2.6 Laboratory measurements

Venous blood samples were collected between 8 and 9 AM following a 24‐hour fast. A standard 75‐g OGTT was used for subjects without DM, and a 100‐g SBMT was used for subjects with DM. Serum N‐terminal propeptide of type I procollagen (s‐PINP) and serum carboxy‐terminal cross‐linking peptide of type I collagen (s‐CTX) were determined by an electrochemiluminescence assay (Elecsys, Roche Diagnostics, Basel, Switzerland). Fasting blood glucose, 2‐hour postload blood glucose, serum triglycerides (TGs), total cholesterol, high‐density lipoprotein cholesterol, low‐density lipoprotein cholesterol, uric acid, calcium, and phosphorus were measured using an autoanalyzer (Cobas 8000 modular analyzer series; Roche Diagnostics, Basel, Switzerland). Glycosylated hemoglobin (HbA1c) was determined by a high‐performance liquid chromatography method using the VARIANT II Hemoglobin Testing System (Tosoh Corporation, Tokyo, Japan).

### 2.7 Statistical analysis

Continuous variables are expressed as the mean ± SD, and categorical variables are expressed as frequency and percentage. Continuous variables were compared using the *t* test (for data conforming to normal distribution and homogeneity of variance) or Wilcoxon test or Kruskal‐Wallis *H* test (for data not conforming to normal distribution or homogeneity of variance). Categorical variables were compared using the chi‐square test. Statistical significance was determined by *p* < 0.05. Binary logistic regression analysis was conducted to test the relationships between the prevalence of LBD/osteoporosis or MOFs and quartiles of SAF‐AGE_age_. A restricted cubic spline nested in logistic models was used to observe the dose–response relationship between SAF‐AGE_age_ and the incidence of LBD/osteoporosis or MOFs. Linear regression analysis was conducted to test the relationships between SAF‐AGEs and bone turnover markers (s‐PINP and s‐CTX). IBM SPSS statistics (version 25.0; SPSS, Inc, Chicago, Illinois) and R (http://www.R-project.org; The R Foundation) software were used to perform all analyses.

## 3 RESULTS

### 3.1 Clinical characteristics

**FIGURE 1:**
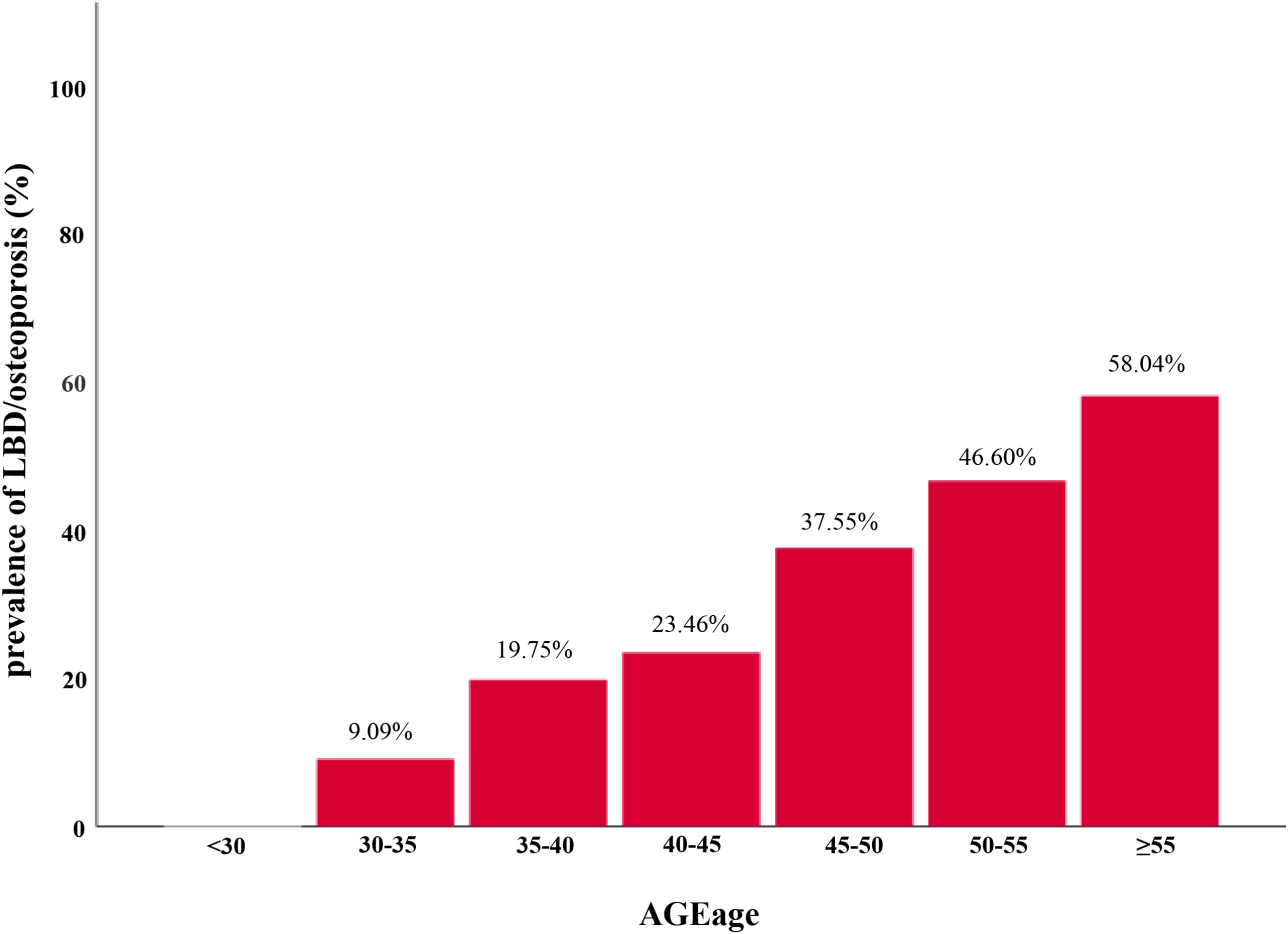
The prevalence of LBD/osteoporosis by categories of SAF‐AGEage levels

Table 1 summarizes the clinical characteristics of the 1214 enrolled participants with T2DM by LBD/osteoporosis status. The overall prevalence of LBD/osteoporosis in these middle‐aged or elderly T2DM patients was 35.7% (n = 434). Mean SAF‐AGE_age_ in groups with and without LBD/osteoporosis was 51.50 ± 9.36 and 45.56 ± 7.62, respectively (*p* < 0.0001). We also found that the incidence of LBD/osteoporosis increased gradually with increasing SAF‐AGE_age_ (Figure 1). T2DM patients with LBD/osteoporosis were significantly more likely to be female and older, to have higher diastolic blood pressure (DBP), fasting plasma glucose (FPG), and serum calcium and phosphorus, and lower WHR (all *p* < 0.05). Moreover, patients with T2DM who currently smoked and drank, had hypertension, took antihypertensives, or lipid‐lowering drugs had a significantly higher incidence of LBD/osteoporosis. There was no significant difference in other characteristics between the two groups (all *p* > 0.05).

**TABLE 1:** Distribution of risk factors in relation to LBD/osteoporosis

	Total N = 1214	Without LBD/osteoporosis n = 780	With LBD/osteoporosis n = 434	*p*
Age, y	64.7 (7.5)	64.24 (7.5)	65.51 (7.5)	**0.005**
Gender male, n (%)	441 (40%)	310 (40%)	131 (30%)	0.002
Menopause (only female), n (%)	757 (97.9%)	459 (97.7%)	298 (98.3%)	0.510
Age at menopause, y	49.57 (4.14)	49.55 (4.21)	49.62 (4.01)	0.843
BMI, kg/m^2^	25.1 (3.3)	25.2 (3.3)	24.9 (3.3)	0.435
WHR	0.78 (0.10)	0.79 (0.10)	0.77 (0.09)	**0.002**
SBP, mmHg	127.70 (29.43)	128.15 (34.72)	126.90 (16.01)	0.481
DBP, mmHg	78.38 (10.06)	79.14 (10.34)	77.01 (9.39)	**<0.0001**
FPG, mmol/L	5.9 (1.7)	5.87 (1.58)	6.08 (1.83)	**0.041**
2‐h PG, mmol/L	6.6 (4.2)	6.45 (3.93)	6.87 (4.54)	0.099
HbA1c (%)	7.81 (1.71)	7.80 (1.72)	7.85 (1.70)	0.579
Diabetes duration, y	10.9 (8.6)	10.50 (8.349)	11.49 (8.98)	0.056
TC, mmol/L	5.4 (1.0)	5.38 (0.97)	5.33 (1.04)	0.412
TGs, mmol/L	1.7 (1.5)	1.74 (1.37)	1.71 (1.78)	0.776
HDL‐C, mmol/L	1.5 (0.4)	1.50 (0.344)	1.48 (0.35)	0.384
LDL‐C, mmol/L	5.4 (3.6)	5.36 (3.39)	5.61 (3.95)	0.241
Ca, mmol/L	2.3 (0.1)	2.25 (0.10)	2.27 (0.12)	**0.002**
P, mmol/L	1.2 (0.1)	1.15 (0.15)	1.17 (0.15)	**0.026**
UA, μmol/L	319.4 (76.4)	322.16 (76.05)	314.56 (76.81)	0.097
AGE_age_	47.7 (8.8)	45.56 (7.62)	51.50 (9.36)	**<0.0001**
Current smoking, n (%)	267 (22.0%)	150 (19.2%)	117 (27.0%)	**0.002**
Current drinking, n (%)	133 (10.9)	189 (24.2%)	131 (30.2%)	**0.024**
Hypertension, n (%)	732 (60.3%)	454 (58.2%)	278 (64.1%)	**0.049**
Hyperlipidemia, n (%)	565 (46.5%)	363 (46.5%)	202 (46.5%)	0.999
Hyperuricemia, n (%)	77 (6.3%)	56 (7.2%)	21 (4.8%)	0.109
Hypertension family history, n (%)	755 (62.1%)	486 (62.3%)	269 (62.0%)	0.911
Diabetes family history, n (%)	690 (56.8%)	455 (58.2%)	236 (54.4%)	0.197
**Antidiabetic agents, n (%)**				
Oral antidiabetes drugs	1016 (83.6%)	657 (84.2%)	359 (82.7%)	0.494
Metformin	602 (49.5%)	400 (51.3%)	202 (46.5%)	0.114
Sulfonylureas	144 (11.9%)	91 (11.7%)	53 (12.2%)	0.778
Thiazolidinediones	8 (0.7%)	7 (0.9%)	1 (0.2%)	0.314
Glinides	10 (0.8%)	8 (1.0%)	2 (0.5%)	0.476
DPP‐IV inhibitors	6 (0.5%)	6 (0.8%)	0	
Glycosidase inhibitors	606 (49.9%)	399 (51.2%)	207 (44.7%)	0.248
SGLT2 inhibitors	0	0	0	
GLP‐1 receptor agonists	12 (1.0%)	9 (1.2%)	3 (0.7%)	0.633
Insulin	309 (25.4)	194 (24.9%)	115 (26.5%)	0.533
**Antihypertension agents, n (%)**	651 (53.6%)	435 (55.8%)	216 (49.8%)	**0.045**
Ca channel blockers	428 (35.3%)	294 (37.7%)	134 (39%)	**0.017**
Angiotensin receptor blockers	208 (17.1%)	158 (20.3%)	50 (11.5%)	**<0.0001**
Angiotensin‐converting enzyme inhibitors	50 (4.1%)	40 (5.1%)	10 (2.3%)	**0.018**
β‐receptor blocker	60 (4.9%)	35 (4.5%)	25 (5.8%)	0.327
Diuretics	10 (0.8%)	6 (0.8%)	4 (0.9%)	0.778
**Lipid‐lowering agents, n (%)**	412 (33.9%)	284 (36.4%)	128 (29.5%)	**0.015**
Statins	356 (29.3%)	246 (31.5%)	110 (25.3%)	**0.023**
Fibrates	8 (0.7%)	5 (0.6%)	3 (0.7%)	0.917
Ca, n (%)	312 (25.7%)	213 (27.3%)	99 (22.8%)	0.086
Calcitriol, n (%)	305 (25.1%)	212 (27.2%)	93 (21.4%)	**0.027**

*Note*: Data are expressed as mean with SD or n (%). The bold values indicates *p* < 0.05.

Abbreviations: AGE, advanced glycation end product; BMI, body mass index; Ca, calcium; DBP, diastolic blood pressure; DPP‐IV, dipeptidyl peptidase IV; FPG, fasting plasma glucose; GLP‐1, glucagon‐like peptide 1; HbA1c, glycosylated hemoglobin; HDL‐C, high‐density lipoprotein cholesterol; LBD, low bone density; LDL‐C, low‐density lipoprotein cholesterol; P, phosphorus; PG, postload blood glucose; SBP, systolic blood pressure; SGLT2, sodium‐glucose cotransporter 2; TC, total cholesterol; TG, triglyceride; UA, uric acid; WHR, waist to hip ratio.

In total, 1101 (90.7%) individuals completed the MOF follow‐up. The overall prevalence of MOFs in middle‐aged or elderly T2DM patients was found to be 10.5% (n = 116). Mean SAF‐AGE_age_ in groups with and without MOFs was 50.19 ± 8.23 and 47.55 ± 8.69, respectively (*p* = 0.002). T2DM patients who smoked had a significantly higher prevalence of MOFs (18.1% vs 8.3%, *p* < 0.001). More detailed characteristics of the two groups are shown in Table 2.

**TABLE 2:** Distribution of risk factors in relation to MOFs

	Total N = 1101	Without MOFs n = 985	With MOFs n = 116	*p*
Age, y	64.71 (7.54)	64.40 (7.40)	67.36 (8.21)	**<0.001**
Gender male, n (%)	395 (35.9%)	357 (36.2%)	38 (32.8%)	0.459
Menopause (only female), n (%)	693 (98.2%)	616 (98.1%)	77 (98.7%)	0.697
Age at menopause, y	49.62 (4.16)	49.57 (4.24)	50.03 (3.45)	0.374
BMI, kg/m^2^	25.10 (3.32)	25.14 (3.30)	24.75 (3.44)	0.237
WHR	0.78 (0.10)	0.78 (0.10)	0.78 (0.08)	0.912
SBP, mm Hg	126.98 (16.45)	126.81 (16.39)	128.43 (16.89)	0.317
DBP, mm Hg	78.46 (9.90)	78.22 (9.78)	80.51 (10.71)	0.**019**
FPG, mmol/L	5.94 (1.68)	5.93 (1.66)	6.05 (1.92)	0.459
2‐h PG, mmol/L	6.64 (4.20)	6.65 (4.13)	6.57 (4.75)	0.855
Diabetes duration, y	10.81 (8.54)	10.77 (8.58)	11.14 (8.19)	0.662
TC, mmol/L	5.36(0.99)	5.36(1.01)	5.39(0.90)	0.736
TGs, mmol/L	1.75(1.58)	1.71(1.34)	2.03(2.90)	**0.039**
HDL‐C, mmol/L	1.49(0.35)	1.49(0.35)	1.48(0.34)	0.781
LDL‐C, mmol/L	5.43(3.61)	5.40(3.57)	5.68(3.97)	0.431
Ca, mmol/L	2.25(0.11)	2.25(0.11)	2.25(0.10)	0.637
P, mmol/L	1.16(0.15)	1.16(0.15)	1.15(0.15)	0.553
UA, μmol/L	318.37 (75.44)	317.10 (74.40)	329.13 (83.30)	0.104
AGE_age_	47.83 (8.68)	47.55 (8.69)	50.19 (8.23)	**0.002**
Current smoking, n (%)	249 (22.6%)	204 (20.7%)	45 (38.8%)	**<0.001**
Current drinking, n (%)	284 (25.8%)	247 (25.1%)	37 (31.9%)	0.112
Hypertension, n (%)	667 (60.6%)	586 (59.5%)	81 (69.8%)	**0.031**
Hyperlipidemia, n (%)	514 (46.7%)	453 (46.0%)	61 (52.6%)	0.178
Hyperuricemia, n (%)	68 (6.2%)	62 (6.3%)	6 (5.2%)	0.635
Hypertension family history, n (%)	682 (61.9%)	605 (61.4%)	77 (66.4%)	0.298
Diabetes family history, n (%)	632 (57.4%)	569 (57.8%)	63 (54.3%)	0.476
**Antidiabetic agents, n (%)**				
Oral antidiabetes drugs	925 (84.0%)	825 (83.8%)	100 (86.2%)	0.496
Metformin	545 (49.5%)	490 (49.7%)	55 (47.4%)	0.635
Sulfonylureas	127 (11.5%)	115 (11.7%)	12 (10.3%)	0.671
Thiazolidinediones	8 (0.7%)	8 (0.8%)	0	
Glinides	9 (0.8%)	8 (0.8%)	1 (0.9%)	
DPP‐IV inhibitors	6 (0.5%)	4 (0.4%)	2 (1.7%)	
Glycosidase inhibitors	553 (50.2%)	489 (49.6%)	64 (55.2%)	0.260
SGLT2 inhibitors	0	0	0	
GLP‐1 receptor agonists	10 (0.9%)	8 (0.8%)	2 (1.7%)	
Insulin	276 (25.1%)	248 (25.2%)	28 (24.1%)	0.807
**Antihypertension agents, n (%)**	591 (53.7%)	521 (52.9%)	70 (60.3%)	0.128
Ca channel blockers	388 (35.2%)	339 (34.4%)	49 (42.2%)	0.095
Angiotensin receptor blockers	189 (17.2%)	166 (16.9%)	23 (19.8%)	0.422
Angiotensin‐converting enzyme inhibitors	43 (3.9%)	41 (4.2%)	2 (1.7%)	0.304
β‐receptor blocker	50 (4.5%)	44 (4.5%)	6 (5.2%)	0.730
Diuretics	9 (0.8%)	9 (0.9%)	0	
**Lipid‐lowering agents, n (%)**	373 (33.9%)	327 (33.2%)	46 (39.7%)	0.165
Statins	322 (29.2%)	280 (28.4%)	42 (36.2%)	0.081
Fibrates	7 (0.6%)	7 (0.7%)	0	
Ca, n (%)	279 (25.3%)	252 (25.6%)	27 (23.3%)	0.589
Calcitriol, n (%)	273 (24.8%)	245 (24.9%)	28 (24.1%)	0.862

*Note*: Data are expressed as mean with SD or n (%). The bold values indicates p<0.05.

Abbreviations: AGE, advanced glycation end product; BMI, body mass index; Ca, calcium; DBP, diastolic blood pressure; DPP‐IV, dipeptidyl peptidase IV; FPG, fasting plasma glucose; GLP‐1, glucagon‐like peptide 1; HDL‐C, high‐density lipoprotein cholesterol; LDL‐C, low‐density lipoprotein cholesterol; MOF, major osteoporotic fracture; P, phosphorus; PG, postload blood glucose; SBP, systolic blood pressure; SGLT2, sodium‐glucose cotransporter 2; TC, total cholesterol; TG, triglyceride; UA, uric acid; WHR, waist to hip ratio.

### 3.2 Association between SAF‐AGE_age_ and LBD/osteoporosis

The univariate binary logistic analysis with SAF‐AGE_age_ as a categorical variable showed a significantly positive relationship between SAF‐AGE_age_ and the risk of LBD/osteoporosis; SAF‐AGE_age_ Q2 versus Q1 showed an odds ratio (OR) of 1.95 (95% CI 1.33–2.87), SAF‐AGE_age_ Q3 versus Q1 was 3.12 (2.14–4.54), and SAF‐AGE_age_ Q4 versus Q1 was 6.11 (4.21–8.88). After adjusting for sex, BMI, WHR, systolic blood pressure (SBP), DBP, FPG, 2‐hour PG, hypertension history, current smoking, current drinking, use of calcium channel blockers (CCBs), angiotensin receptor blockers (ARBs), angiotensin‐converting enzyme inhibitors (ACEIs), statins, calcium, or calcitriol the ORs (95% CI) were 2.02 (1.34–3.03), 3.63 (2.44–5.39), and 6.51 (4.34–9.78), respectively **(**Table 3).

**TABLE 3:** Association of SAF‐AGE_age_ with LBD/osteoporosis among patients with type 2 diabetes

	AGE_age_				Per SD increase	*p* for trend
	≤41.66	41.67–46.37	46.38–52.59	≥52.60		
**Total**						
No. of patients	303	304	304	303		
No. of cases	53	89	121	171		
OR	1.00	1.95 (1.33–2.87)	3.12(2.14–4.54)	6.11(4.21–8.88)	2.11 (1.84–2.43)	<0.001
Multivariable‐adjusted OR	1.00	2.02 (1.34–3.03)	3.63(2.44–5.39)	6.51(4.34–9.78)	2.22(1.90–2.58)	<0.001
**Male**						
No. of patients	109	109	105	118		
No. of cases	17	23	30	61		
OR	1.00	1.45(0.72–2.89)	2.19(1.11–4.22)	5.79(3.08–10.89)	1.09 (1.07–1.12)	<0.001
Multivariable‐adjusted OR	1.00	1.45(0.70–3.03)	2.96(1.45–6.05)	7.40(3.62–15.13)	2.48(1.89–3.26)	<0.001
**Female**						
No. of patients	194	195	199	185		
No. of cases	36	66	91	110		
OR	1.00	2.25(1.41–3.59)	3.70(2.34–5.84)	6.44 (4.04–10.26)	1.09 (1.07–1.11)	<0.001
Multivariable‐adjusted OR	1.00	2.34(1.42–3.86)	4.15(2.54–6.78)	6.48(3.88–10.83)	2.17(1.79–2.64)	<0.001
**BMI < 25.0**						
No. of patients	149	161	156	158		
No. of cases	26	47	67	91		
OR	1.00	1.95(1.13–3.56)	3.56(2.10–6.04)	6.43(3.79–10.89)	1.09 (1.07–1.11)	<0.001
Multivariable‐adjusted OR	1.00	2.13(1.20–3.76)	4.49(2.55–7.88)	6.34(3.59–11.21)	2.09(1.69–2.58)	<0.001
**BMI ≥ 25.0**						
No. of patients	153	142	148	145		
No. of cases	27	41	54	80		
OR	1.00	1.89(1.09–3.29)	2.68(1.57–4.57)	5.74(3.38–9.75)	1.09 (1.07–1.12)	<0.001
Multivariable‐adjusted OR	1.00	1.84(1.02–3.33)	2.85(1.61–5.04)	6.62(3.65–12.00)	2.37(1.89–2.98)	<0.001
**Current smoking (yes)**						
No. of patients	70	76	59	62		
No. of cases	17	26	29	45		
OR		1.62 (0.79–3.34)	3.01 (1.42–6.37)	8.25 (3.78–18.02)	2.38 (1.74–3.26)	<0.001
Multivariable‐adjusted OR		1.38 (0.61–3.14)	3.56 (1.55–8.15)	6.49 (2.68–15.69)	2.26 (1.58–3.22)	<0.001
**Current smoking (no)**						
No. of patients	233	228	245	241		
No. of cases	36	63	92	126		
OR		2.09 (1.32–3.31)	3.29 (2.12–5.11)	6.00 (3.88–9.27)	2.12 (1.82–2.49)	<0.001
Multivariable‐adjusted OR		2.19 (1.36–3.55)	3.32 (2.09–5.27)	5.86 (3.69–9.31)	2.12 (1.79–2.51)	<0.001
**Current drinking (yes)**						
No. of patients	89	89	65	77		
No. of cases	20	29	31	51		
OR		1.67 (0.86–3.25)	3.15 (1.57–6.31)	6.77 (3.41–13.44)	2.29 (1.73–3.02)	<0.001
Multivariable‐adjusted OR		1.76 (0.83–3.73)	4.24 (1.91–9.38)	7.82 (3.57–17.13)	2.44 (1.77–3.35)	<0.001
**Current drinking (no)**						
No. of patients	214	215	239	226		
No. of cases	33	60	90	120		
OR		2.12 (1.32–3.42)	3.31 (2.10–5.22)	6.21 (3.94–9.78)	2.12 (1.80–2.50)	<0.001
Multivariable‐adjusted OR		2.23 (1.35–3.66)	3.14 (1.95–5.04)	5.53 (3.412–8.97)	2.04 (1.71–2.43)	<0.001
**Diabetes family history (yes)**						
No. of patients	192	183	164	151		
No. of cases	34	52	63	87		
OR	1.00	1.85 (1.13–3.01)	2.90 (1.78–4.71)	6.32 (3.87–10.33)	2.27 (1.87–2.77)	<0.001
Multivariable‐adjusted OR	1.00	1.80(1.07–3.04)	3.25(1.94–5.46)	6.60(3.81–11.41)	2.40(1.92–3.00)	<0.001
**Diabetes family history (no)**						
No. of patients	111	121	140	152		
No. of cases	19	37	58	84		
OR	1.00	2.13 (1.14–3.99)	3.43 (1.88–6.23)	5.98 (3.32–10.77)	1.99 (1.63–2.43)	<0.001
Multivariable‐adjusted OR	1.00	2.33(1.20–4.51)	4.11(2.18–7.75)	6.87(3.63–13.03)	2.13(1.71–2.66)	<0.001

*Note*: Adjusted for BMI, waist to hip ratio, systolic blood pressure, diastolic blood pressure, fasting plasma glucose, 2‐h postload blood glucose, hypertension, current smoking, current drinking, calcium channel blockers, angiotensin receptor blockers, angiotensin‐converting enzyme inhibitors, statins, calcium, and calcitriol.

Abbreviations: AGE, advanced glycation end product; BMI, body mass index; LBD, low bone density; OR, odds ratio; SAF, skin autofluorescence.

**FIGURE 2:**
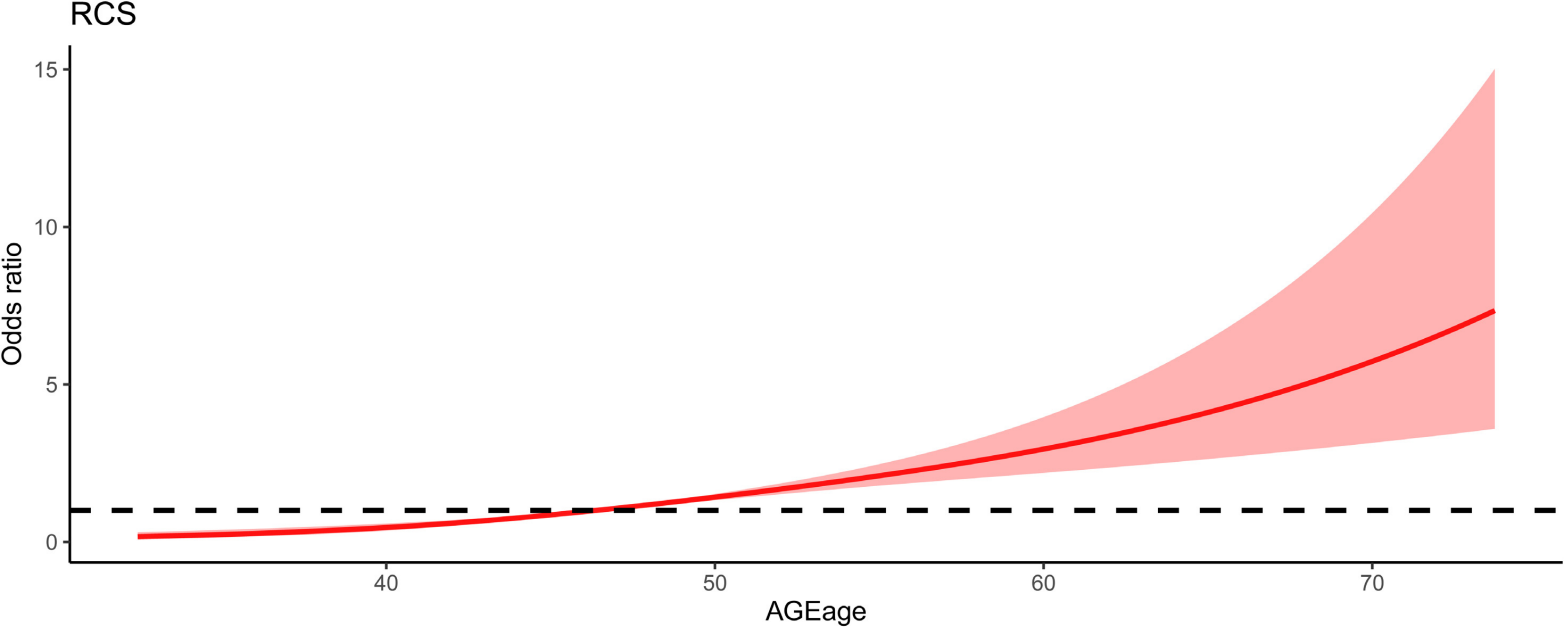
The spline association between SAF‐AGEage levels and LBD/osteoporosis

Logistic analysis with SAF‐AGE_age_ as a continuous variable also showed a significant association, with an OR (95% CI) of 2.11 (1.84–2.43) per SD increase of SAF‐AGE_age_ in the crude model and 2.22 (1.90–2.58) in the multivariate adjusted model (both *p* for trend <0.001) (Table 3). When restricted cubic splines were used in the logistic model with SAF‐AGE_age_ as a continuous variable, a graded positive relationship was also observed between the incidence of LBD/osteoporosis and SAF‐AGE_age_ (Figure 2).

Stratified analyses were conducted according to sex, BMI, current smoking, current drinking, and family history of diabetes. The results were similar across all subgroups and in accordance with that identified in the whole group (Table 3). No interactions were observed when tested by subgroup analyses.

### 3.3 Association between SAF‐AGE_age_ and MOFs

The ORs (95% CI) of MOFs across SAF‐AGE_age_ quartiles were 1.12 (0.58–2.17), 2.24 (1.24–4.04), and 2.51 (1.40–4.49) in the crude model and 1.02 (0.52–2.02), 2.42 (1.32–4.46), and 2.70 (1.48–4.91) in the multivariate‐adjusted model including sex, BMI, DBP, hypertension, current smoking, current drinking, CCB, statins, and serum TGs (Table 4).

**TABLE 4:** Association of SAF‐AGE_age_ with MOFs among patients with type 2 diabetes

	AGE_age_				Per SD increase	*p* for trend
	≤41.73	41.74–46.44	46.45–52.28	≥52.29		
**Total**						
No. of patients	276	276	274	275		
No. of cases	18	20	37	41		
OR	1.00	1.12 (0.58–2.17)	2.24 (1.24–4.04)	2.51 (1.40–4.49)	1.31 (1.10–1.56)	0.003
Multivariable‐adjusted OR	1.00	1.02 (0.52–2.02)	2.42 (1.32–4.46)	2.70 (1.48–4.91)	1.39 (1.15–1.67)	0.001
**Male**						
No. of patients	98	101	91	105		
No. of cases	6	9	16	7		
OR	1.00	1.50 (0.51–4.39)	3.27 (1.22–8.77)	1.10 (0.36–3.38)	1.11 (0.82–1.50)	0.510
Multivariable‐adjusted OR	1.00	1.46 (0.48–4.45)	4.67 (1.62–13.44)	1.23 (0.38–4.00)	1.17 (0.85–1.63)	0.338
**Female**						
No. of patients	178	175	183	170		
No. of cases	12	11	21	34		
OR	1.00	0.93 (0.40–2.16)	1.79 (0.85–3.76)	3.46 (1.72–6.94)	1.46 (1.17–1.82)	0.001
Multivariable‐adjusted OR	1.00	0.81 (0.33–1.95)	1.85 (0.86–3.96)	3.79 (1.84–7.82)	1.56 (1.23–1.97)	<0.001
**BMI < 25.0**						
No. of patients	137	147	144	140		
No. of cases	9	9	20	27		
OR	1.00	0.93 (0.36–2.41)	2.29 (1.01–5.23)	3.40 (1.53–7.53)	1.50 (1.17–1.93)	0.002
Multivariable‐adjusted OR	1.00	0.82 (0.31–2.17)	2.26 (0.96–5.33)	3.56 (1.56–8.16)	1.60 (1.22–2.10)	0.001
**BMI ≥ 25.0**						
No. of patients	138	128	130	135		
No. of cases	9	10	17	14		
OR	1.00	1.22 (0.48–3.09)	2.16 (0.93–5.03)	1.66 (0.69–3.97)	1.18 (0.91–1.52)	0.214
Multivariable‐adjusted OR	1.00	1.30 (0.50–3.40)	2.47 (1.03–5.97)	1.92 (0.78–4.74)	1.23 (0.94–1.61)	0.126
**Current drinking (yes)**						
No. of patients	79	82	53	70		
No. of cases	8	8	8	13		
OR	1.00	0.96 (0.34–2.70)	1.58 (0.55–4.50)	2.02 (0.79–5.22)	1.19 (0.89–1.61)	0.246
Multivariable‐adjusted OR	1.00	0.86 (0.29–2.55)	1.53 (0.51–4.56)	1.86 (0.67–5.16)	1.23 (0.87–1.72)	0.238
**Current drinking (no)**						
No. of patients	197	194	221	205		
No. of cases	10	12	29	28		
OR	1.00	1.23 (0.52–2.92)	2.82 (1.34–5.96)	2.96 (1.40–6.27)	1.40 (1.13–1.74)	0.002
Multivariable‐adjusted OR	1.00	1.17 (0.48–2.87)	3.04 (1.41–6.53)	3.47 (1.60–7.51)	1.49 (1.19–1.88)	0.001
**Current smoking (yes)**						
No. of patients	65	74	50	60		
No. of cases	10	10	13	12		
OR		0.86 (0.33–2.22)	1.93 (0.77–4.87)	1.38 (0.55–3.47)	1.21 (0.87–1.67)	0.260
Multivariable‐adjusted OR		0.93 (0.35–2.52)	1.98 (0.73–5.40)	1.56 (0.58–4.20)	1.26 (0.89–1.80)	0.199
**Current smoking (no)**						
No. of patients	211	202	224	215		
No. of cases	8	10	24	29		
OR		1.32 (0.51–3.42)	3.05 (1.34–6.94)	3.96 (1.76–8.87)	1.42 (1.15–1.76)	0.001
Multivariable‐adjusted OR		1.22 (0.46–3.24)	3.40 (1.47–7.84)	4.53 (1.99–10.32)	1.51 (1.21–1.88)	<0.001
**LBD or osteoporosis (yes)**						
No. of patients	49	83	107	155		
No. of cases	4	8	9	21		
OR		1.20 (0.34–4.21)	1.03 (0.30–3.53)	1.76 (0.58–5.41)	1.09 (0.82–1.44)	0.564
Multivariable‐adjusted OR		1.14 (0.30–4.29)	0.99 (0.28–3.55)	1.82 (0.57–5.83)	1.17 (0.85–1.60)	0.332
**LBD or osteoporosis (no)**						
No. of patients	227	193	167	120		
No. of cases	14	12	28	20		
OR		1.01 (0.46–2.24)	3.07 (1.56–6.03)	3.04 (1.48–6.27)	1.69 (1.30–2.20)	<0.001
Multivariable‐adjusted OR		0.89 (0.39–2.02)	3.74 (1.84–7.62)	3.78 (1.77–8.07)	1.84 (1.39–2.43)	<0.001

*Note*: Adjusted for sex, BMI, diastolic blood pressure, hypertension, current smoking, current drinking, calcium channel blockers, statins, and serum triglycerides other than the variable for stratification.

Abbreviations: AGE, advanced glycation end product; BMI, body mass index; LBD, low bone density; MOF, major osteoporotic fracture; OR, odds ratio; SAF, skin autofluorescence.

**FIGURE 3:**
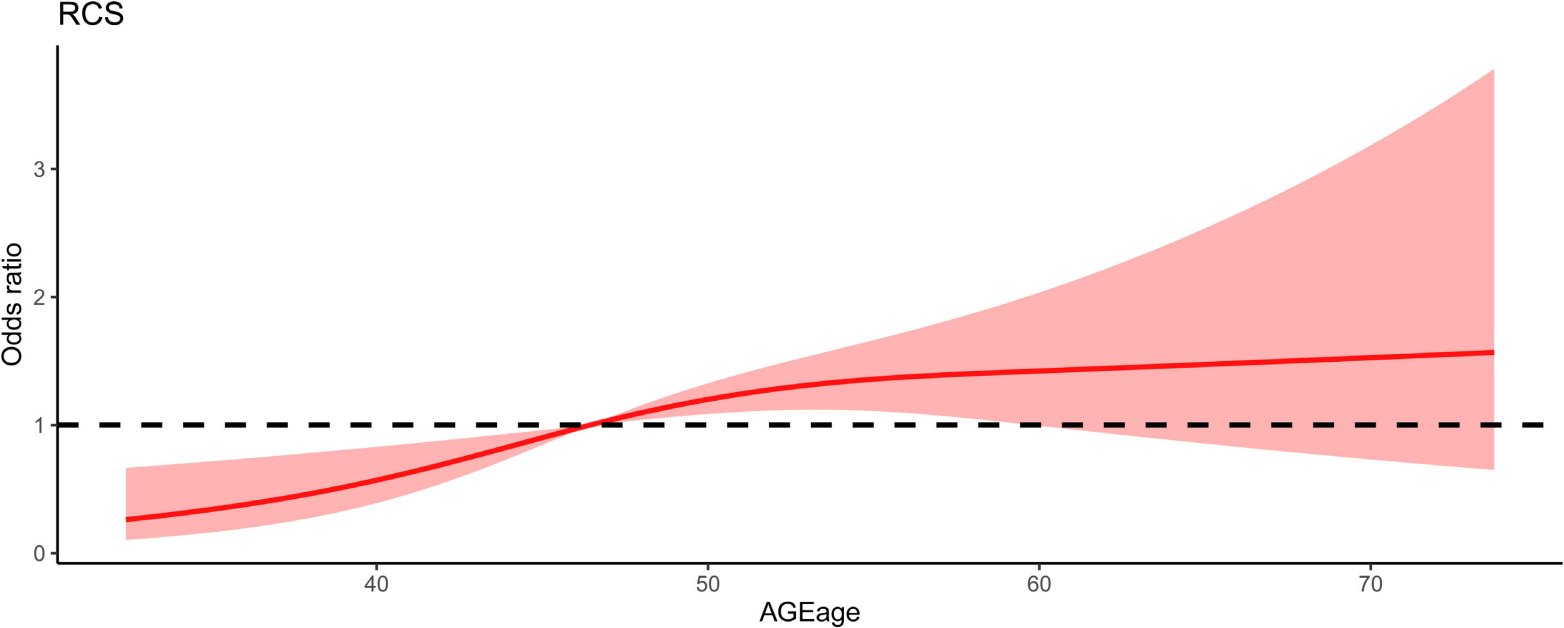
The spline association between SAF‐AGEage levels and MOFs

Logistic analysis with SAF‐AGE_age_ as a continuous variable also showed a significant association with an OR (95% CI) of 1.31 (1.10–1.56) per SD increase of SAF‐AGE_age_ in the crude model (*p* for trend = 0.003) and 1.39 (1.15–1.67) for the multivariate adjusted model (*p* for trend <0.001) (Table 4). When restricted cubic splines were used in the logistic model with SAF‐AGE_age_ as a continuous variable, a positive relationship was also observed between the incidence of MOFs and SAF‐AGE_age_ (Figure 3).

When stratified according to sex, SAF‐AGE_age_ was significantly associated with MOFs only in females; OR (95% CI) 1.56 (1.23–1.97) per SD increase of SAF‐AGE_age_ in the multivariate‐adjusted model (*p* for trend <0.001). In males the OR (95% CI) was 1.17 (0.85–1.63) (*p* for trend = 0.338) (Table 4). When stratified by BMI, current smoking, current drinking, and LBD/osteoporosis, there was a significant association only in patients who did not smoke, did not consume alcohol, did not have LBD/osteoporosis, or had a BMI < 25 (OR 1.51, CI 1.21–1.88, *p* < 0.001; OR 1.49, CI 1.19–1.88, *p* = 0.001; OR 1.84, CI 1.39–2.43, *p* < 0.001; and OR 1.60, CI 1.22–2.10, *p* = 0.001), but not in patients who smoked, consumed alcohol, had LBD/osteoporosis, or a BMI ≥ 25 (OR 1.26, CI 0.89–1.80, *p* = 0.199; OR 1.23, CI 0.87–1.72, *p* = 0.238; OR 1.17, CI 0.85–1.60, *p* = 0.332; and OR 1.23, CI 0.94–1.61, *p* = 0.126). Subgroup analyses showed no interaction effects between SAF‐AGE_age_ and sex, BMI, current smoking, or current drinking in fully adjusted models.

### 3.4 Association between SAF‐AGEs and s‐PINP or s‐CTX

Linear regression analysis showed that higher SAF‐AGEs were associated with a higher level of s‐PINP and s‐CTX, with an increase in s‐PINP of 1.09 ng/ml (95% CI, 0.31–1.87; *p* = 0.006) and s‐CTX of 5.60 ng/L (95% CI, 1.35–10.64; *p* = 0.011) per 10 units increase in SAF‐AGEs in the univariate model. In the model adjusted for sex, BMI, HbA1c, and age, the association of SAF‐AGEs with s‐PINP was attenuated with an OR (95% CI) reduced to 1.02 (0.24–1.80, *p* = 0.011), but the association of SAF‐AGEs with s‐CTX was strengthened with an OR (95% CI) increased to 6.30 (1.77–10.83, *p* = 0.006) (Table 5).

**TABLE 5:** Association of SAF‐AGEs (per 10 units) with s‐PINP and s‐CTX among patients with type 2 diabetes

	s‐PINP, ng/mL		s‐CTX, ng/mL	
	OR (95% CI)	*p*	OR (95% CI)	*p*
Univariate model	1.09 (0.31–1.87)	0.006	5.60 (1.35–10.64)	0.011
Adjusted model	1.02 (0.24–1.80)	0.011	6.30 (1.77–10.83	0.006

*Note*: Adjusted model by sex, body mass index, HbA1c, and age.

Abbreviations: AGE, advanced glycation end product; HbA1c, glycosylated hemoglobin; OR, odds ratio; SAF, skin autofluorescence; s‐CTX, serum carboxy‐terminal cross‐linking peptide of type I collagen; s‐PINP, serum N‐terminal propeptide of type I procollagen.

## 4 DISCUSSION

In the present study, we report that SAF‐AGE_age_ is closely associated with the incidence of LBD/osteoporosis in patients with T2DM. Moreover, with the categories of increasing SAF‐AGE_age_, the incidence of LBD/osteoporosis gradually increased. This trend existed among patients of different sexes, BMI, smoking and drinking status, and family history of diabetes. There are few published studies examining the relationship between BMD and AGEs in patients with T2DM. Raška et al found no relationship between serum circulating receptors of AGEs and BMD in postmenopausal women with T2DM.^17^ Yavuz et al found that osteoporotic patients with T2DM have a higher SAF‐AGEs level, which is consistent with our results.^18^ A series of studies have found that AGEs can influence the structure and function of type I collagen, an important component of bone mass.^19^, ^20^, ^21^ AGEs may change the osteoblasts and osteoclast capacity for bone turnover by inducing chronic inflammation, thereby degrading bones and changing BMD.^22^

We found a positive association of SAF‐AGEs with s‐PINP and s‐CTX. It is well‐known that the rate of bone turnover in T2DM patients is lower compared with healthy individuals.^23^, ^24^, ^25^ In vitro experiments have found that AGEs can adversely affect osteoclast and osteoblast differentiation and function and decrease bone turnover.^26^, ^27^, ^28^ Lamb et al. found that the plasma endogenous secretory receptor for AGEs (esRAGE) is positively related to PINP in old men with or without T2DM.^29^ Eckhardt et al reported that in a type 2 diabetes mouse model, AGEs can change bone turnover by binding to their corresponding receptors.^30^ Rubin et al studied the association between SAF‐AGEs and s‐PINP, s‐CTX, bone‐specific alkaline phosphatase (bone ALP), and tartrate‐resistant acid phosphatase 5b (TRACP5b) in adults with type 1 diabetes mellitus.^31^ They found a positive relationship between SAF‐AGEs and all bone turnover markers, but this association was attenuated in adjusted models.^31^

We also identified a relationship between SAF‐AGE_age_ and MOFs (mainly hip and vertebral fractures). However, there was no difference in BMD and bone turnover markers (s‐PINP and s‐CTX) between patients with and without MOFs. In a T‐score‐stratified analysis for MOFs, we found an association between SAF‐AGEs and MOFs in patients without LBD or osteoporosis, but not in those with LBD or osteoporosis. It appears contradictory that SAF‐AGE_age_ is negatively associated with BMD and nonlinearly associated with MOFs, but that there is no relationship between BMD and MOF. Lower BMD and active bone turnover are two significant predictors of bone fractures. Therefore, other mechanisms may exist to cause bone fractures by AGEs in T2DM patients. It has been demonstrated that patients with T2DM have a higher incidence of osteosarcopenia and sarcopenia and a lower trabecular bone score (TBS).^32^, ^33^, ^34^ One animal model study found that a high‐AGE diet can destroy the vertebral microarchitecture resulting in vertebral fractures.^35^ Other animal studies have found that the biomechanical properties of the bone matrix are altered by the accumulation of AGEs in the bone of diabetic mice.^25^, ^36^, ^37^ An in vitro study by Maghami et al found that AGEs can influence the crack‐growth trajectory of human cortical bone by promoting crack formation.^38^ Piccoli et al found that the accumulation of AGEs in bone tissue can destroy bone microarchitecture in older women with T2DM by increasing the space between trabeculae and decreasing volumetric BMD.^39^ Choi Y. J. et al demonstrated that T2DM patients with higher urinary pentosidine have lower TBS.^40^ Furst et al found that SAF‐AGEs are associated with the bone material strength index (BMSi) in T2DM patients.^25^ These studies may explain the contradiction observed in the present study, by implying the complexity of AGE effects on bone strength.

Higher serum pentosidine in T2DM patients with vertebral fractures has been reported in some studies.^41^, ^42^ Although we used different variables (SAF‐AGE_age_ and serum pentosidine) and different fracture sites, we showed similar results. However, in contrast with our results, the Rotterdam study found no association between SAF‐AGE_age_ and MOFs or vertebral fractures in T2DM patients.^15^ Several factors may underlie these contradictory findings. First, the time of SAF measurement was different. In our study, SAF measurement preceded the MOF follow‐up. Second, the inclusion criteria were somewhat different; we excluded individuals with conditions such as end‐stage kidney disease and malignancy that may affect bone metabolism, thereby reducing possible sources of confounding. Third, the assessed fracture sites (MOFs of the hip, vertebra wrist, or humerus versus vertebral fractures only in patients with T2DM) were different. Finally, the sample size of T2DM patients was different. Although the Rotterdam study had a large total sample size, the number of subjects with T2DM was relatively small (353 vs 1214 in our study), thereby suggesting that our results are more generalizable to the population.

Following stratification by smoking and drinking status, we found an association between SAF‐AGEs and MOFs in nonsmokers and nondrinkers, but not in current smokers and current drinkers. Some studies have demonstrated the association between current smoking or alcohol consumption and SAF‐AGEs^43^, ^44^, ^45^, ^46^ but we did not observe this association. Some potential factors may exist. First, the majority of smokers and drinkers in our study were older men. Second, there is marked heterogeneity in cigarette and alcohol type and quantity between different studies, which may lead to inconsistent results. Finally, the mechanism by which cigarettes and alcohol affect bone fractures is complex and comprehensive.^47^, ^48^ Following the stratified analysis by sex, we found that SAF‐AGEs were associated with MOFs in women but not in men, which was consistent with the Rotterdam study general population results.^15^

There are some limitations to the present study. First, the loss to follow‐up for MOFs was 9.3% due to incorrect contact details, death, no response, etc. Second, the enrolled individuals were from two communities located in urban Beijing. The results obtained from these individuals may be generalizable to at least the wider middle‐aged and elderly Beijing and Chinese T2DM population. However, further work is required to confirm the generalizability of our findings.

In conclusion, we found that SAF‐AGE_age_ is positively associated with the prevalence of LBD/osteoporosis in patients with T2DM. We also found a positive association between SAF‐AGEs and s‐PINP and s‐CTX. There was an association between SAF‐AGE_age_ and the incidence of MOFs that was independent of BMD. Larger prospective studies are still needed to explore the effects of AGEs on bone fractures in T2DM patients.

## AUTHOR CONTRIBUTIONS

Hongyan Liu and Guoqi Wang analyzed the data. Hongyan Liu was a major contributor in writing the manuscript. Guoqi Wang performed the statistical analysis. Ting Wu and Jia Hu searched data. Weijun Gu and Yiming Mu were the superior advisors. They gave final approval of the version to be published. All authors read and approved the final manuscript.

## FUNDING INFORMATION

This study was supported by the National Key R&D Program of China (project no. 2016YFC1305200).

## CONFLICT OF INTEREST

The authors declare no conflicts of interest in this work.

## PATIENT CONSENT STATEMENT

Written informed consent was obtained from all participants prior to participation.

## PERMISSION TO REPRODUCE MATERIAL FROM OTHER SOURCES

There is no material from other sources.

## Data Availability

The datasets used to support this study are not freely available in view of participants' privacy protection.
